# Assessing cognitive dysfunction in Parkinson's disease: An online tool to detect visuo‐perceptual deficits

**DOI:** 10.1002/mds.27311

**Published:** 2018-02-23

**Authors:** Rimona S. Weil, Dietrich S. Schwarzkopf, Bahador Bahrami, Stephen M. Fleming, Ben M. Jackson, Tristam J. C. Goch, Ayse P. Saygin, Luke E. Miller, Katerina Pappa, Ivanna Pavisic, Rachel N. Schade, Alastair J. Noyce, Sebastian J. Crutch, Aidan G. O'Keeffe, Anette E. Schrag, Huw R. Morris

**Affiliations:** ^1^ Dementia Research Centre, Institute of Neurology, University College London London UK; ^2^ Department of Molecular Neuroscience Institute of Neurology, University College London London; ^3^ Institute of Cognitive Neuroscience, University College London London UK; ^4^ Department of Experimental Psychology London UK; ^5^ School of Optometry & Vision Science, Faculty of Medical & Health Sciences University of Auckland Auckland New Zealand; ^6^ Wellcome Centre for Human Neuroimaging, University College London London UK; ^7^ Damn Fine Ltd London UK; ^8^ Department of Cognitive Science University of California San Diego California USA; ^9^ Wolfson Institute of Preventive Medicine, Barts and the London School of Medicine and Dentistry Queen Mary University of London London UK; ^10^ Department of Statistical Science University College London London UK; ^11^ Department of Clinical Neurosciences Royal Free Campus Institute of Neurology, University College London London UK

**Keywords:** Parkinson's disease, vision, perception, hallucinations, signal detection theory

## Abstract

**Background:** People with Parkinson's disease (PD) who develop visuo‐perceptual deficits are at higher risk of dementia, but we lack tests that detect subtle visuo‐perceptual deficits and can be performed by untrained personnel. Hallucinations are associated with cognitive impairment and typically involve perception of complex objects. Changes in object perception may therefore be a sensitive marker of visuo‐perceptual deficits in PD.

**Objective:** We developed an online platform to test visuo‐perceptual function. We hypothesised that (1) visuo‐perceptual deficits in PD could be detected using online tests, (2) object perception would be preferentially affected, and (3) these deficits would be caused by changes in perception rather than response bias.

**Methods:** We assessed 91 people with PD and 275 controls. Performance was compared using classical frequentist statistics. We then fitted a hierarchical Bayesian signal detection theory model to a subset of tasks.

**Results:** People with PD were worse than controls at object recognition, showing no deficits in other visuo‐perceptual tests. Specifically, they were worse at identifying skewed images (*P* < .0001); at detecting hidden objects (*P* = .0039); at identifying objects in peripheral vision (*P* < .0001); and at detecting biological motion (*P* = .0065). In contrast, people with PD were not worse at mental rotation or subjective size perception. Using signal detection modelling, we found this effect was driven by change in perceptual sensitivity rather than response bias.

**Conclusions:** Online tests can detect visuo‐perceptual deficits in people with PD, with object recognition particularly affected. Ultimately, visuo‐perceptual tests may be developed to identify at‐risk patients for clinical trials to slow PD dementia. © 2018 The Authors. Movement Disorders published by Wiley Periodicals, Inc. on behalf of International Parkinson and Movement Disorder Society.

Dementia affects up to 50% of people with Parkinson's disease (PD) within 10 years of diagnosis^1^ with great societal and financial impacts. Converging evidence shows that PD patients with involvement of visual processing are at highest risk of PD dementia.^2^, ^3^ However, current measures of visuo‐perceptual function are poorly sensitive (eg, copying intersecting pentagons^4^ or clock drawing^5^ or require trained personnel and are time consuming to perform (such as the Vision Object and Space Perception battery^6^ or Benton's Judgment of Line Orientation^7^, ^8^.

Visual hallucinations affect 40% to 50% of people with PD^9^, ^10^ and typically involve perception of complex objects, implicating object processing pathways.^10^ We reasoned that tests of object recognition would be most likely to detect deficits in PD. In the healthy brain, object recognition includes visual search^11^ and object invariance.^12^ It is not well studied in PD, with reports limited to detection of embedded patterns^13^ and facial emotion recognition.^14^

Deficits in visuo‐perceptual processing could potentially be caused by bottom‐up sensory deficits, or top‐down factors, such as response bias, a tendency to report signals as present or absent. The framework of signal detection theory allows us to test which is most affected in PD. The theory proposes that perceptual performance is determined by sensitivity of a system to signal relative to background noise, known as d‐prime (*d'*), and bias to report signal presence, described as criterion, *c*.^15^ Although criterion shifts may be driven by changes at either a perceptual or decisional locus,^16^ changes in sensitivity are modelled as changes in signal‐to‐noise ratio, indicating a selective effect on perceptual processing.^15^ Given the involvement of visual processing in PD, we hypothesized that people with PD would show impaired perceptual sensitivity with relative preservation of criterion.

We therefore developed an online test of visuo‐perception in PD. We hypothesised that (1) visuo‐perceptual deficits in PD could be detected using online tests, (2) object perception tasks would be preferentially affected, and (3) differences in performance would be caused by changes in perception rather than response bias.

## Materials and Methods

### Participants

**Figure 1:**
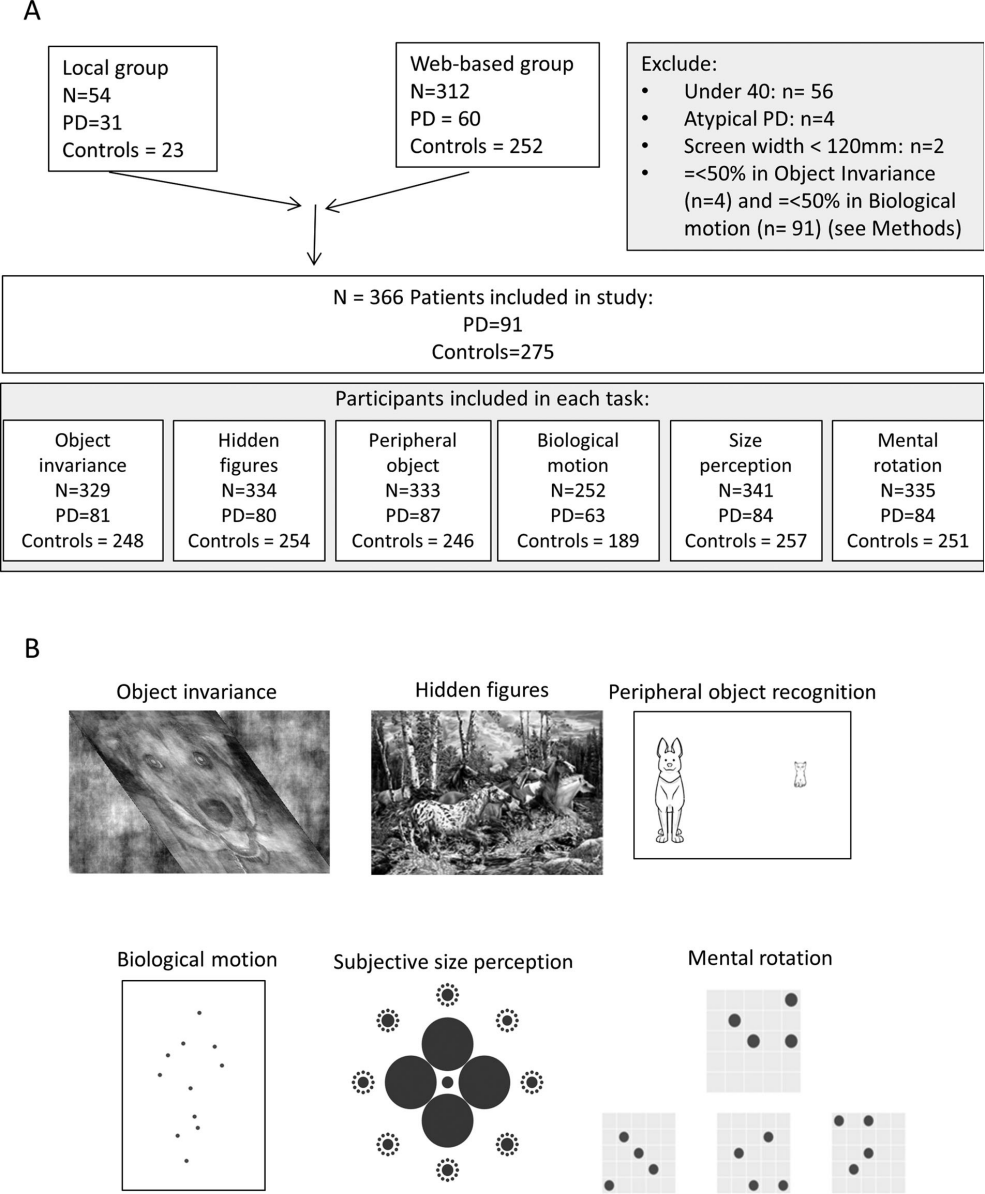
(**A**) Patient recruitment and inclusion in the study, showing the number of patients and controls in each online task. (**B**) Online visuo‐perceptual tasks: **Object invariance.** Example skewed image. A dog is shown here. On each trial, an image of a cat or dog was shown for 280 milliseconds. Participants were then shown a choice screen, where they reported whether they had seen a cat or a dog using the computer mouse (3000 milliseconds response time, 24 trials). **Hidden figures.** The image contains 22 horses, 7 of which are not hidden, the rest are formed within the background features. Participants used their computer mouse to click on all the horses they could find. A maximum of 6 minutes and 75 clicks was allowed to reduce “blind” clicking across the entire image. Image produced by Steven M. Gardner and used here with permission. **Peripheral object detection.** Two images of animals were shown: one at fixation the other in the periphery. Presentation time was 280 milliseconds, followed by a choice screen where participants indicated whether the 2 presented animals were the same or different in identity. The image at fixation was smaller to enforce central fixation (24 trials). **Biological motion.** Participants were shown a moving point‐light walker, either with dots at the position of the major joints of a person moving, or with the position of the dots scrambled so that no percept of a person is formed. Participants indicated whether they had seen a person or scrambled moving dots (3000 milliseconds response time, 24 trials). **Subjective size perception.** In the classical form of the Ebbinghaus illusion, 2 identical circles are surrounded by smaller or larger inducers. This causes a perceived difference in the size of the central circles. Here, we modified this illusion by surrounding the larger inducing circles by 8 test circles that were each surrounded by 12 inducers. One of the test circles matched the central target circle in diameter. The others differed by a pseudorandom amount, drawn from a normal distribution around the diameter of the reference circle. The position of the identical test circle differed on each trial. Participants selected the test circle that matched the central circle in size using the computer mouse (15 trials). **Mental rotation.** Participants selected the grid in the lower row that matched the grid in the top row, but rotated (24 trials).

Participants accessed the study in 2 ways: a local group accessed the website at our study center, and a web‐based group accessed the website independently (Fig. 1A). Patients in the local group were recruited from PD clinics at the National Hospital for Neurology and Neurosurgery and Royal Free Hospital, London, UK. Inclusion criteria were clinically diagnosed PD (Queen Square Brain Bank criteria, early to mid‐stage disease [Hoehn & Yahr 1‐3]). Exclusion criteria included confounding neurological or psychiatric disorders, eye disease, or dementia. Participants with PD continued their usual levodopa therapy. Age‐matched controls were recruited from university databases and unaffected spouses. Participants in the web‐based group were recruited from PD clinics, support groups, and a national newspaper article. We excluded participants aged 40 or younger and those with atypical PD (Fig. 1A). Duplicate attempts were removed (highest‐scoring performance retained). All participants gave informed consent on an online consent form, with additional written informed consent for the local group. The study was approved by the Queen Square Research Ethics Committee.

### Assessment of Local Participants

The severity of symptoms was assessed using the MDS‐UPDRS. Visual acuity was measured using a 6‐m chart and converted to decimal acuity. Contrast sensitivity was measured using a Pelli‐Robson chart (SSV‐281‐PC; http://www.sussex-vision.co.uk). Cognition was assessed using the Mini‐Mental State Examination and Montreal Cognitive Assessment.

### Online Assessments

Participants accessed the website (https://vision-in-parkinsons.co.uk/) using a Dell latitude 3340 laptop (Dell (Round Rock, Texas, USA)) (local group) or their own devices. Participants completed a clinical questionnaire on the website, providing demographic information and disease status and indicating the presence or absence of visual hallucinations (Table 1).

**Table 1:** Demographics of participants across all tasks

Demographics of all participants				
Clinical characteristics	Patients	Controls	*t* / or χ^2^ (df)	*P*
N	91	275	NA	NA
Male/female	54/37	68/207	36.9 (1)	<.0001
Age, y, (SD), (range)	66.0 (8.8), (41‐88)	61.7 (9.5), (41‐85)	4.0 (164)	.00010
Disease duration, y (SD)	4.85 (3.89)	NA	NA	NA
N with hallucinations/without	15 /76	0/275	NA	NA
Demographics of local patients				
N	31	23	NA	NA
Male/female	18/13	10/13	1.13 (1)	.29
Age (years) (SD), (range)	66.9 (7.3), (49‐80)	67.3 (10.7), (41‐80)	−0.15 (36.9)	.88
Disease duration (years) (SD)	4.9 (3.8)	NA	NA	NA
N with hallucinations/without	5/26	0/23	NA	NA
MoCA	27.2 (2.8) (n = 30)	28.1 (2.0), (n = 22)	−1.3 (50.0)	.19
MMSE	29.2 (1.0) (n = 28)	29.5 (0.59)	−1.3 (44.3)	.19
Snellen visual acuity	0.99 (0.26)	0.97 (0.28)	0.23 (43.3)	.82
Contrast sensitivity	1.76 (0.19) (n = 20)	1.78 (0.16) (n = 14)	−0.26 (30.7)	.80
MDS‐UPDRS 1	8.74 (5.4)	2.0 (2.4)	6.2 (43)	<.0001
MDS‐UPDRS 2	9.10 (4.6)	0.3 (0.7)	10.4 (31.9)	<.0001
MDS‐UPDRS 3	20.5 (9.0)	3.4 (5.0)	8.8 (48.7)	<.0001
MDS‐UPDRS 4	1.16 (2.8)	0 (0)	2.3 (30)	.026
MDS‐UPDRS total	39.6 (16.5)	5.9 (5.8)	10.5 (39.8)	<.0001
Hoehn & Yahr	1.47 (0.63)	NA	NA	NA

All participants tested, unless stated otherwise. MMSE, mini‐mental state examination; MoCA, Montreal Cognitive Assessment; SD, standard deviation; NA, not applicable; MDS‐UPDRS, Movement Disorder Society Unified Parkinson's Disease Rating Scale; 1 = nonmotor symptoms; 2 = motor symptoms; 3 = objective motor score; 4 = motor complications.

To mitigate against screen variation, the tasks were internally controlled for differences in aspect ratios, resolutions, and viewing distances. We avoided tasks dependent on contrast sensitivity requiring screen gamma correction. Attempts to participate using a mobile phone were redirected to a larger monitor. Images were rescaled according to screen resolution and monitor size with participant‐inputted information to calculate mm/pixel. The online tests took approximately 45 minutes and were generally well received by participants.

### Tests of Visuo‐Perceptual Function

The participants underwent practice sessions prior to each task. A fixation cross between trials (0.75° visual angle, 1500 milliseconds) encouraged central fixation. Short presentation times prevented eye movement confounds.

### Object Recognition Tests (Fig. 1B)

#### 1 Object Invariance

This probes ability to recognise objects at extreme angles and may be linked with infero‐temporal cortex activity.^17^ We recently showed^18^ that this process is impaired in PD. Whether deficits can be detected using a short online test is not known.

Stimuli were prepared using MATLAB 2014a (https://uk.mathworks.com/).^18^ A total of 50 images (25 cats, 25 dogs) from an open online database (http://www.kaggle.com) were converted to grayscale and Fourier transformed. The phase matrix of each image was combined with white noise and the average magnitude matrix of the whole stimulus set using an inverse Fourier transform.^19^ The resulting image was sheared using an affine matrix transformation (3 levels of skew: 0, 1.4, and 2.3 arbitrary units, order pseudorandomized). The images were scaled relative to screen width and subtended 4° × 13°. On each trial the skewed image was presented centrally for 450 milliseconds followed by a choice screen (3000 milliseconds, 24 trials). Participants scoring ≤ 50% at the lowest difficulty level were excluded (PD, n = 2; controls, n = 2).

#### 2 Hidden Figures

Misperception of objects in PD^10^ may be caused by top‐down processes, where patients are primed to see objects more readily, or to impaired object detection. We tested these 2 competing theories using object detection within a complex scene.^11^

The stimulus was an image depicting 22 horses (SM Gardner, with permission [http://www.gardnergallery.com/], 7 horses not hidden, remainder formed within surrounding scenery; Fig. 1B). Image size 13° × 17° (scaled to screen height). The participants clicked on every horse they found (maximum 75 clicks, 6 minutes). Total number of horses found, repeat selections, incorrect selection of nonhorse areas, and total time were recorded.

#### 3 Peripheral Object Recognition

People with PD show reduced contrast sensitivity in the periphery.^20^ We used a matching task to test peripheral object detection.

Images of a cat, dog, or mouse were shown in pairs (1 central, 1 at 81% screen width, side pseudorandomized, 450 milliseconds, followed by choice screen, 24 trials; Fig. 1B). Image identity was either matched or different. To enforce central fixation, the images were unequal in size (central image height 2.5°, peripheral image height 7.5°).

#### 4 Biological Motion

Displaying a dot at the position of joints of a moving person evokes the perception of a person moving.^21^ Neuroimaging implicates superior temporal sulcus^22^ in this percept. Biological motion perception may be affected in PD.^23^ Whether this can be detected using online tests is not known.

Stimuli were point‐light walkers (12 blue dots on a white background^24^. Motion‐matched noise dots (0, 10, or 30) were added, providing 3 levels of difficulty. Control gifs were generated using the point‐light walkers, with dot position and motion scrambled. Figure height subtended 5°.

On each trial a point‐light walker or scrambled motion was selected pseudorandomly (displayed for 2 cycles, 3000 milliseconds, followed by choice screen, 24 trials). Participants scoring ≤ 50% at lowest difficulty level were excluded (PD, n = 23; controls, n = 68).

### Other Visuo‐Perceptual Tests

#### 1 Subjective Size Perception

A central circle surrounded by smaller circles appears larger than an identically sized circle surrounded by larger circles.^25^ Behavioral and neuroimaging studies suggest that this is mediated by the primary visual cortex (V1).^26^ We hypothesized that if pathological processes associated with PD involve V1, the magnitude of this illusion might be reduced, and patients would perform better than controls at this task.

The stimulus was adapted from the classic illusion to maximise comparisons with fewer trials. A central small circle (the “target,” diameter 20 px, 0.5°), was surrounded by 4 large inducers (diameter 90 px, 22.5°). This was surrounded by 8 peripheral “test” circles, with varying diameter, each surrounded by 12 small inducers (diameter 6 px, 1.5°; Fig. 1B). On every trial, 1 of the 8 test circles was identical in diameter to the target circle (with varying position). The diameter of the 7 test circles varied pseudorandomly in a log‐normal distribution around the exact diameter of the target circle.

Participants selected the test circle matching the central target circle (15 trials). The ratio of test circle to target circle indicated the magnitude of illusion for that participant. Geometric mean ratio of selected circles was calculated and transformed back into log space.

#### 2 Mental Rotation

This is the ability to match rotated objects and may involve mid‐level visual processing regions such as V3.^27^ PD patients carrying microtubule associated protein tau (MAPT) polymorphisms show deficits in this process.^28^ Whether deficits can be seen in PD in general is not known.

Stimuli consisted of a reference 5 × 5 grid, above 3 test grids. Within the reference grid were 4 blue circles (pseudorandom positions). Test grids were rotated ±90° or ± 180°. In 2 test grids, a blue circle was moved to an adjacent position. Participants selected the grid that was identical to the reference grid, but rotated (24 trials).

### Online Estimate of Visual Acuity

A Taylor's‐E optotype was presented, surrounded on 4 sides by outward‐pointing triangles (Supplemental Fig. 1). The participants clicked the triangle beside the open side of the “E.” The size of the next optotype was determined using an adaptive staircase converging onto the participant's acuity (step sizes 0.05 acuity, 20 steps).^29^ Visual acuity was calculated as mean of the last 4 trials.

### Tapping Test

An online tapping test (based on the BRAIN test,^30^ validated for assessment of bradykinesia in PD) assessed bradykinesia as objective support of PD diagnosis. This is a keyboard‐tapping task based on alternate finger‐tapping between 2 computer keys spaced 15‐cm apart. It reliably differentiates between individuals with PD and controls. Participants used the index finger of 1 hand to press the “S” and “;” keys for 30 seconds, for each hand. The number of key taps in 30 seconds was recorded. Scores less than 10 and ≥100 were excluded.^30^ The average score for both hands was calculated.

### Statistical Analysis

Performance was compared between groups using 2‐tailed Welch's *t* tests, Mann–Whitney tests for nonnormally distributed data, or repeated measures analyses of variance (where tasks had more than 1 difficulty level). We accounted for confounding influences of age by adjusting for age and separately examining differences between groups when the oldest PD patients were removed until the groups were age matched.

We used linear regression to examine effects of factors on task performance. We then accounted for differences in age and gender by including these variables as covariates in additional regression models for effect of PD on task performance. Where tasks included more than 1 difficulty level, the most discriminatory level was used, defined as greatest effect size, followed by highest significance level. *P* < .05, Bonferroni corrected for multiple comparisons (n = 6 comparisons, significance < .0083), was accepted as the threshold of statistical significance. Statistical analysis was performed using R (https://www.r-project.org/).

### Signal Detection Analysis

To assess whether perceptual deficits in PD are reflected either by changes in perception and/or a bias in decisions and reporting, we used the signal detection theory framework. Robust estimation of parameters of the signal detection model typically requires large numbers of trials per participant, which would not be feasible using an online testing system. Hierarchical models allow robust estimates of group‐level parameters in cases with large samples, but few trials per participant, as here. Unlike fixed‐effects models that collapse data across participants, a hierarchical Bayesian approach is the correct way to combine information about within‐ and between‐subject uncertainty, such that group‐level parameters are less influenced by single‐subject fits that have high degree of uncertainty. In turn, hierarchical modelling mutually constrains subject‐level parameter estimation because of the shared dependence on group‐level parameters.^31^

We fit the hierarchical Bayesian signal detection theory model to tasks that employed a 2‐alternative‐forced‐choice design. Responses were sorted into hits, false alarms, misses, and correct rejections. For each participant, detection sensitivity (*d'*) and criterion (c) were calculated separately:

$$\begin{array}{l} d\prime \;=\;z\left(H\right)–z\left(\text{FA}\right)\\ c=\;-0.5\;x\left(z\left(H\right)\;+z\left(FA\right)\right) \end{array}$$

where *z* indicates inverse of the cumulative normal distribution, *H* hit rate, and *FA* false alarm rate. We additionally fitted a hierarchical Bayesian signal detection model^32^ to obtain posterior distributions of group‐level sensitivity (*d'*) and bias (*c*) parameters. We used Markov Chain Monte Carlo using Gibbs sampling implemented in JAGS (Just Another Gibbs Sampler) in R^33^ to draw samples from the posterior distributions. We used uninformative (high variance) prior distributions on the group‐level estimates of d′ and criterion when fitting calibration data (after JAGS convention, variances are written as precisions, or the reciprocal of the variance, denoted as *λ*):

$$\begin{array}{l} \mu_{d^{\prime }}∼N\left(0,0.001\right)\\ \mu_{c}∼N\left(0,0.001\right)\\ \lambda_{d^{\prime }}∼Gamma\left(0.001,0.001\right)\\ \lambda_{c}∼Gamma\left(0.001,0.001\right)\\ d^{\prime }∼\;N\left(\mu_{d^{\prime }},\;\lambda_{d^{\prime }}\right)\\ c∼\;N\left(\mu_{c},\;\lambda_{c}\right) \end{array}$$

Here, *N*() refers to a standard normal distribution with given mean and precision; λ() refers to a gamma distribution parameterized by shape and rate parameters. $\mu_{d^{\prime }}$ and $\mu_{c}$ are group means for *d'* and criterion, respectively.

JAGS was called with 2000 adaptation steps, 5000 burn‐in samples, and 50,000 effective samples; 3 chains for each parameter were run. Convergence of chains was assessed visually and using the potential scale‐reduction statistic $\hat{R}$. For each task, we fitted the model twice, once for PD patients, once for controls. Posterior distributions for each group‐level parameter returned by JAGS, in each participant group, were employed for Bayesian inference.^34^ To assess differences between controls and people with PD we calculated the probability that the difference between the 2 parameters was larger than zero, P_θ_( $\mu_{d^{\prime },\;controls}$ *‐* $\mu_{d^{\prime },\;Parkinson\prime s}$ *>0)*, where high probability indicates strong evidence in favor of a difference. We denote these probabilities as P_θ_ to distinguish them from classic *P* values.

## Results

### Demographics

A total of 428 participants accessed the website across the 2 groups. We excluded 62 participants based on exclusion criteria of age, screen width, and atypical PD (Fig. 1A). The remainder were 91 participants with PD and 275 controls, with slight variation in participant numbers in each task (Fig. 1A). Mean age PD = 66.0 ± 8.8 years; controls = 61.7 ± 9.5 years, average disease duration = 4.85 ± 3.89 years (Table 1). Of the participants, 15 reported visual hallucinations. In the local group there was no difference in cognition, visual acuity, or contrast sensitivity between people with PD and controls (Table 1). Although we did not directly assess web‐based participants, there were no differences in demographic details or in visuo‐perceptual tests between patients in local and web‐based groups (Supplemental Tables 1 and 2).

### Performance in Object Recognition Tests

**Figure 2:**
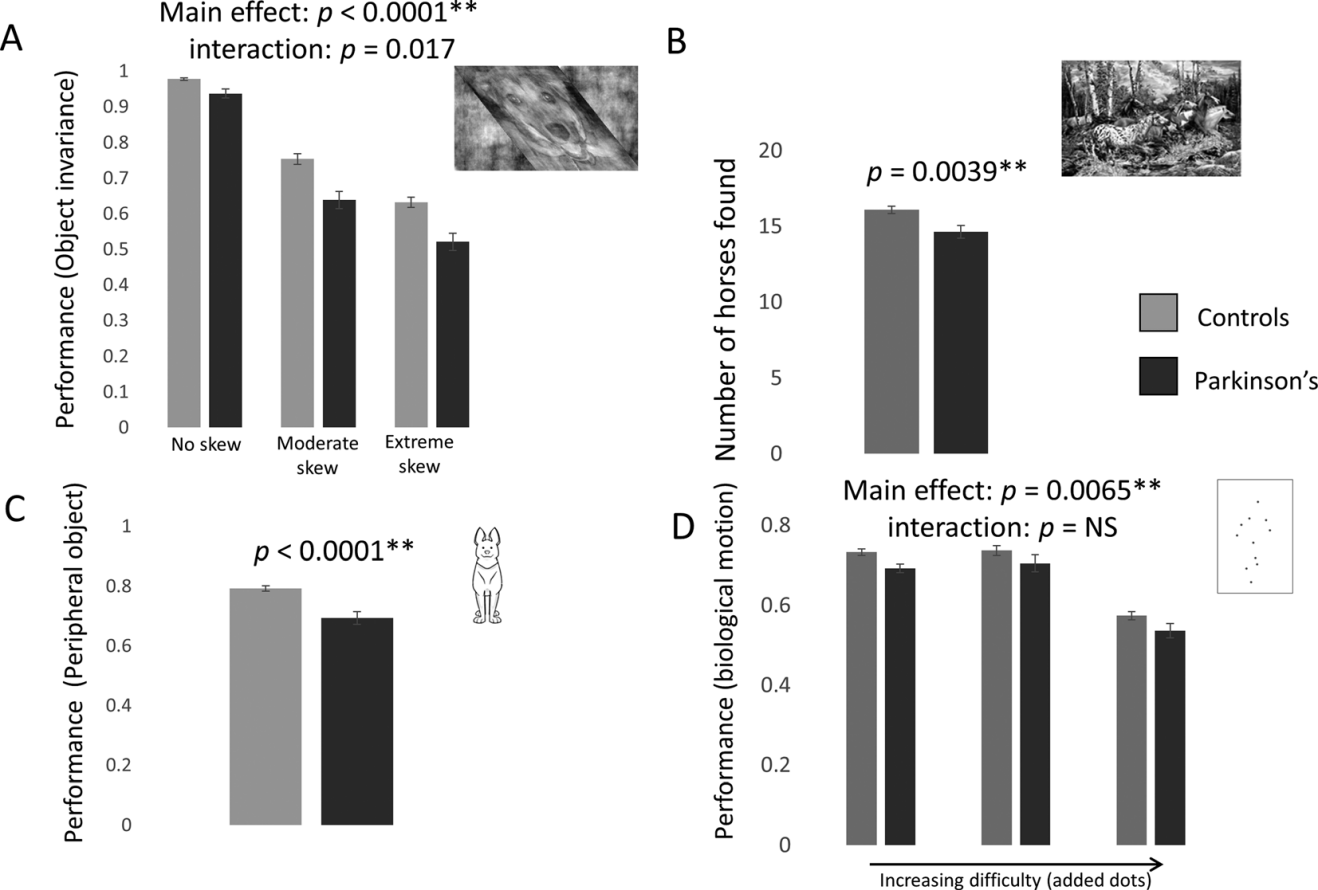
Results. (**A**) Object invariance. Performance in the object invariance test (identifying skewed animals) at 3 levels of skew for controls (light gray) and people with PD (dark gray). Main effect Parkinson's disease group and difficulty, both *P* < .0001. Error bars are standard error of the mean in all panels. ** Significant after Bonferroni correction in all panels. (**B**) Hidden figures. Number of horses found by controls (light gray) and people with PD (dark gray). (**C**) Peripheral object detection. Performance in matching animals presented at fixation and in peripheral visual field for controls (light gray) and people with PD (dark gray). (**D**) Biological motion. Performance in detecting biological motion at 3 levels of difficulty (additional moving dots) for controls (light gray) and people with PD (dark gray). Main effect Parkinson's disease group and difficulty, both *P* < .0001.

People with PD were worse at all tasks involving object recognition. They were worse at object invariance (main effect PD: *F*_1,327_ = 24.8, *P* < .0001; main effect difficulty: *F*_2,654_ = 433, *P* < .0001). The interaction between presence of PD and difficulty tended to significance at a Bonferroni‐corrected level: *F*_2,654_ = 4.1, *P* = .017 (Fig. 2A, Table 2).

**Table 2:** Performance in each of the online tasks for participants with Parkinson's disease and controls

Task n	Subsection of task	Parkinson's, mean (SD)	Controls, mean (SD)	*t* (degrees of freedom)	*P*
Object invariance PD: n = 81 HC: n = 248	Skew level 1	0.94 (0.1)	0.98 (0.1)	W = 8375^a^	.0012^b^
	Skew level 2	0.64 (0.2)	0.75 (0.2)	−4.0 (143)	<.0001^b^
	Skew level 3	0.52 (0.2)	0.63 (0.2)	−3.9 (142)	<.0001^b^
Hidden Figures PD: n = 80 HC: n = 254	Number horses found	14.6 (3.8)	16.1 (3.9)	−2.9 (136)	.0039^b^
	Background selected	20.8 (15)	20.3 (15)	0.29 (133)	.77
	Repeat horses	12.0 (8.9)	11.5 (9.8)	0.44 (144)	.66
	Total time (s)	216.6 (135)	208.4 (108)	0.49 (112)	.62
Peripheral object recognition PD: n = 87 HC: n = 246	Performance	0.69 (0.2)	0.79 (0.1)	−4.33 (119)	<.0001^b^
Biological motion PD: n = 63 HC: n = 189	Level 1	0.69 (0.1)	0.73 (0.1)	−2.9 (139)	.0038^b^
	Level 2	0.70 (0.2)	0.74 (0.2)	−1.3 (107)	.20
	Level 3	0.54 (0.2)	0.57 (0.1)	−1.8 (101)	.075
Subjective size perception PD: n = 84 HC: n= 257	Performance (log ratio)	‐0.06 (0.03)	‐0.07 (0.04)	1.86 (165)	.064
Mental rotation PD: n = 84 HC: n = 251	Performance	0.84 (0.1)	0.85 (0.1)	−1.0 (159)	.32
Visual acuity	Decimal acuity	0.74 (0.3)	0.82 (0.3)	−2.5 (146)	.013
Tapping test PD: n = 90 HC: n = 267	Number of taps in 30 seconds	42.2 (15)	52.7 (18)	−5.3(187)	<.0001^b^

HC, control; PD, Parkinson's disease; s, seconds; SD, standard deviation.

a Mann–Whitney test performed as data for skew level 1 not normally distributed.

b Significant after Bonferroni correction.

People with PD found fewer horses; mean PD = 14.6 ± 3.8; controls = 16.1 ± 3.9, *t*_136_ = ‐2.9, *P* = .0039 (Fig. 2B, Table 2). There was no difference in the repeat selection of horses, in the selection of nontarget regions (pareidolia), or total time between the groups (Table 2).

PD patients were worse at recognizing animals in peripheral vision than controls (mean PD = 0.69 ± 0.19, controls = 0.79 ± 0.14, *P* < .0001; Fig. 2C) and worse at detecting biological motion (main effect PD: *F*_1,250_ = 7.5, *P* = .0065; main effect difficulty: *F*_2,500_ = 128, *P* < .0001; Fig. 2D). There was no interaction between presence of PD and difficulty (*F*_2,500_ = 0.046, *P* = .96; Table 2).

### Performance in Other Visuo‐Perceptual Tests

We found no difference in subjective size perception or mental rotation in PD when compared with controls (Table 2).

### Effects of Other Factors on Performance

Performance in object invariance and peripheral object recognition was worse with age, but when adjusted for, we continued to see an effect of PD (Supplemental Table 3). Hidden figures performance was also worse with age, but the effects of PD were no longer significant when adjusted for age. There was no effect of age on biological motion, subjective size perception, or mental rotation.

Object invariance and peripheral object recognition was worse in the men than in the women, especially in people with PD. When we adjusted for gender, we continued to see a strong effect of PD.

In biological motion, female controls tended to perform worse than male controls, an effect not seen in PD. When we examined the overall relationship between gender and performance, no significant relationship was seen. Performance in hidden figures tended to be worse in men (with and without PD), and the effect of PD was no longer significant when adjusted for gender. The men also showed a slightly lower effect for subjective size perception. There was no effect of gender on mental rotation (Supplemental Table 3).

We found no relationship between visuo‐perceptual performance and disease duration or presence of hallucinations (Supplemental Tables 4 and 5).

There was no relationship between performance in visuo‐perceptual tasks and visual acuity, with the exception of object invariance, which showed a trend (that disappeared when adjusted for age) and peripheral object recognition (Supplemental Table 4). Effects of PD on peripheral object recognition persisted when adjusted for acuity (estimate = 0.10, standard error = 0.02, *P* < .0001), showing differences were not solely caused by acuity deficits.

Repeat analyses excluding trials with no responses had no effect on our findings (Supplemental Table 6), indicating that worse performance was not driven by higher number of missed trials.

### Other Online Measures

The presence of PD trended to correlation with visual acuity (*R* ^2^ = 0.017, *P* = .013), and this was not significant when adjusted for age (estimate = 0.049 ± 0.030), *t* = 1.7, *P* = .098).

Mean number of taps was lower in PD when compared with controls (Table 2), providing supportive evidence that the online group suffered from PD.

### Hierarchical Modelling of Signal Detection Theory Parameters

**Figure 3:**
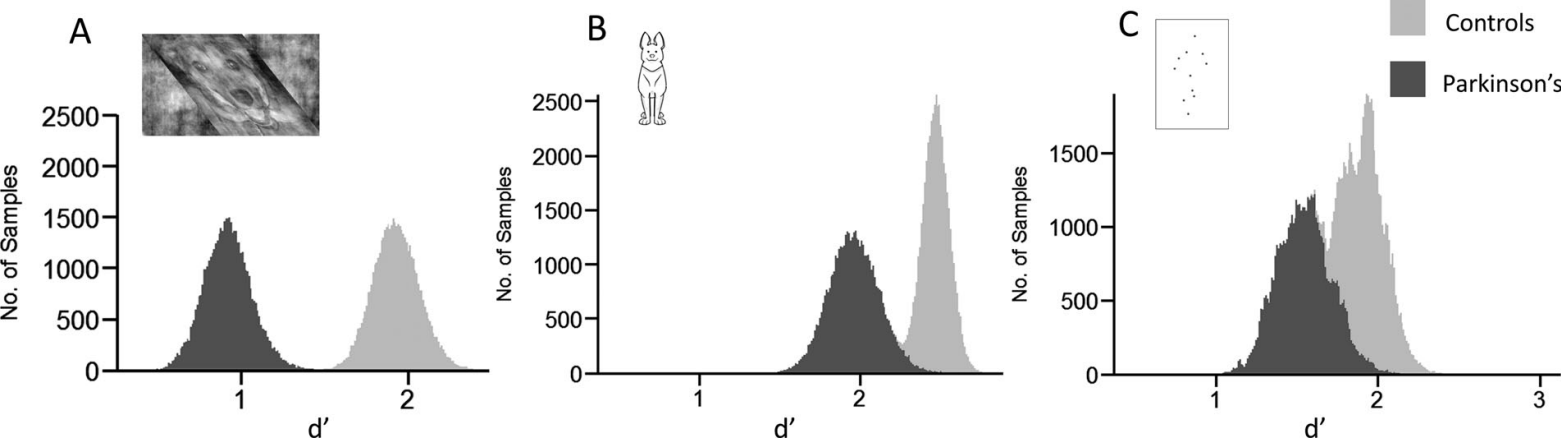
Posterior densities of people with PD (dark gray in each plot) and age‐matched controls (light gray in each plot) for perceptual sensitivity (d‐prime, d') in object invariance (**A**), peripheral object detection (**B**), and biological motion (**C**). Increased separation of the density plots reflects confidence in difference in perceptual sensitivity between the 2 participant groups.

We fit a hierarchical Bayesian signal detection model to participants' data in object invariance, peripheral object recognition, and biological motion tasks. These tasks all adopted a 2‐alternative‐forced‐choice response, allowing us to specify false alarms as well as hits. This enabled us to obtain group‐level posterior distributions of sensitivity (d') and bias (c) separately for controls and PD patients. Trials with no responses were excluded from this analysis (Supplemental Table 7). This approach revealed significant effects of PD on perceptual sensitivity (d') in all 3 tasks. Conversely, there were no group differences in detection criterion, a measure of general bias in reporting presence or absence of signal, regardless of sensory input (Fig. 3, Supplemental Table 8).

## Discussion

Our online visuo‐perceptual platform detected deficits in people with PD when compared with controls. This suggests that these or similar tasks could potentially be used for disease stratification to identify patients with visuo‐perpetual deficits.

We show that performance in object recognition, tested using a variety of tasks, is preferentially affected in PD, suggesting higher level visual processing deficits. Conversely, subjective size perception, which involves the earliest stages of cortical visual processing^35^ and mental rotation, which may involve mid‐level visual processing (eg, V3^27^, were not affected. Until recently, object recognition has not been well studied in PD. Studies using measures such as the Vision Object and Space Perception battery have shown inconsistent findings.^36^, ^37^ Recognition of embedded figures may be affected,^13^ and emotion recognition, a specialized form of object recognition, is impaired.^14^ We recently showed that object invariance is affected in PD,^18^ and deficits in biological motion in PD have recently been shown.^23^ Here we take this further by demonstrating that object recognition is affected ahead of other aspects of visuo‐perception and can be detected using online tests.

The involvement of higher level visuo‐perceptual regions is consistent with structural neuroimaging studies showing atrophy of parieto‐occipital and occipito‐temporal regions in PD.^38^

The signal detection approach allowed us to show that these visuo‐perceptual deficits are caused by differences in perceptual sensitivity rather than response bias. We provide new evidence that people with PD have lower perceptual sensitivity for object recognition and that effects are not explained by differences in response bias.

We found that visuo‐perceptual deficits correlated with age, but not disease duration. This is consistent with literature showing that cognitive involvement in PD is more prevalent in older patients rather than a function of disease duration.^1^, ^39^ This may relate to a synergistic relationship between α‐synuclein and Alzheimer's pathology that is more prevalent in older patients: α‐synuclein promotes neurofibrillary tau tangle formation,^40^ and β‐amyloid induces pathological α‐synuclein phosphorylation.^41^

There are some methodological limitations to this study. Three tasks required a time‐limited response. However, when we repeated analyses excluding “no response” trials we continued to show worse performance in PD, suggesting differences cannot be purely attributed to slower responses.

The number of people with hallucinations was relatively small, which may have contributed to the lack of differences between people with visual hallucinations. The lower prevalence rate of hallucinations here reflects the earlier disease‐stage, community‐based nature of our population when compared with other studies showing higher prevalence rates.^9^, ^10^

We detected object‐level recognition deficits even in patients with no known dementia and without visual hallucinations, suggesting our tests can detect cognitive involvement before people become symptomatic. Visual hallucinations usually involve animate hallucinations. Whether object‐recognition deficits are specific to animate versus inanimate objects needs to be tested in future studies.

Limited clinical data were collected for people participating only using the online platform. We were therefore unable to determine effects of disease severity and motor phenotype on visuo‐perceptual performance. Furthermore, we relied on patients' reports of PD diagnosis, although these were supported by scores on a motor dexterity task. Future work could use a version of this online task in combination with clinical data, including formal diagnostic criteria of PD and medication doses, to directly address this.

We found both age and male gender were associated with poorer performance on some of the visuo‐perceptual tasks, particularly the hidden figures test, where these effects persisted when adjusted for the presence of PD. How these factors interact with the presence of PD is not yet known and could be specifically addressed to better understand the progression of the disease.

All patients were taking their routine medications which may have influenced performance. We previously showed that object invariance is not related to levodopa dose,^18^ and PET imaging studies suggest cholinergic rather than dopaminergic metabolism is more closely related to cognitive function in PD.^42^ This could be addressed in future work.

Finally, our data include a range of patients with varying disease duration, age, and phenotype. This heterogeneity may underlie some variation shown here. To determine whether performance in visuo‐perceptual tasks has prognostic validity, we need longitudinal analyses, alongside standard measures of disease severity and cognitive function and to determine clinically useful cut‐offs using these standard measures as comparisons. Future studies may examine the sensitivity of these online measures in cohorts at risk of PD, such as those with rapid eye movement sleep disorder or anosmia.

In summary, we show that visuo‐perceptual deficits in PD can be detected with short online tests, that tasks of object recognition are preferentially affected, and that perceptual sensitivity deficits, rather than shifts in response bias, underlie these changes. This platform could be developed in future as a potential tool for stratification for patients in the earlier stages of disease for clinical trials aimed at slowing or preventing dementia in PD.

## Author Roles

1) Research project: A. Conception, B. Organization, C. Execution; 2) Statistical Analysis: A. Design, B. Execution, C. Review and Critique; 3) Manuscript: A. Writing of the first draft, B. Review and Critique.

R.S.W.: 1A, 1B, 1C, 2A, 2B, 3A

D.S.S.: 1A, 3B

B.B.: 1A

S.M.F.: 2A, 3B

B.M.J.: 1C

T.J.C.G.: 1C

A.P.S.: 1B

L.E.M.: 1B

K.P.: 1C

I.P.: 1C

R.N.S.: 1C

A.J.N.: 3B

S.J.C.: 1B, 3B

A.O.K.: 2A, 2B, 2C

A.E.S.: 2C, 3B

H.R.M.: 1A, 3B

## Financial disclosures of all authors (for the preceding 12 months)

RSW: No disclosures. DSS: No disclosures. BB: No disclosures. SMF: No disclosures. BMJ: No disclosures. TJCG: No disclosures. APS: No disclosures. LEM: No disclosures. KP: No disclosures. IP: No disclosures. RNS: No disclosures. AJN: Reports support from Prothena pharmaceuticals. SJC: No disclosures. AOK: No disclosures. AES: Reports personal fees from Medtronic and AstraZeneca. HRM: Reports personal fees from Teva, AbbVie, Boehringer Ingelheim, and GlaxoSmithKline.

## Supporting information

Additional Supporting Information may be found in the online version of this article at the publisher's website.

Supplementary Information

Supplementary Information
