# Supplementary figures and images for: Assessing cognitive dysfunction in Parkinson's disease: An online tool to detect visuo‐perceptual deficits
Source: Mov Disord. 2018 Feb 23;33(4):544–53. doi: 10.1002/mds.27311 (PMC5901022; doi:10.1002/mds.27311)

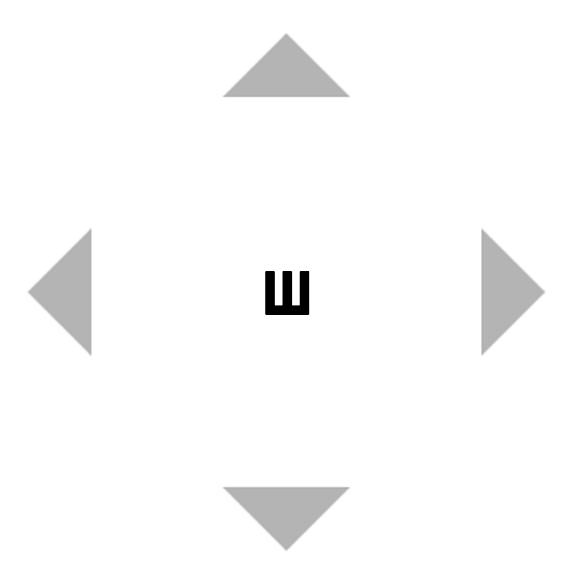

Supplement: Supplementary file 1 — Supplementary Information [file MDS-33-544-s001.tif]
