# Supplementary material for: Assessing cognitive dysfunction in Parkinson's disease: An online tool to detect visuo‐perceptual deficits
Source: Mov Disord. 2018 Feb 23;33(4):544–53. doi: 10.1002/mds.27311 (PMC5901022; doi:10.1002/mds.27311)
Supplement: Supplementary file 2 — Supplementary Information [file MDS-33-544-s002.docx]

**Supplemental Table 1. Comparison of local and web-based participants**

|  | Local Patients | Local  Controls | Web  patients | Web  controls | T / **or** χ^2^ | *p* |
| --- | --- | --- | --- | --- | --- | --- |
| N | 31 | 23 | 60 | 252 | 35.9 | <0.0001 |
| N with/without  hallucinations | 5/26 | 0/23 | 10/50 | 0/252 | 0.0043 | 0.95 |
| Male / Female | 18/13 | 10/13 | 36/24 | 58/194 | PD: 0.032  HC: 4.74 | PD: 0.9  HC: 0.03 |
| Age (years) (SD) (range) | 66.9 (7.3)(49-80) | 67.3 (10.7) (41-80) | 65.5 (9.5) (41-88) | 61.2 (9.2) (41-85) | PD: 0.74  HC: 2.66 | PD: 0.5  HC: 0.01 |
| Disease duration (years)(sd) | 4.9 (3.8) (0.5-16) | NA | 4.8 (4.0) (0.5-22) | NA | -0.09 | 0.9 |

**Supplemental Table 2: Performance in each of the online tasks for participants with Parkinson’s disease and controls**

| **Task** | **n**  **PD/ controls**  **local** | **Mean (SD)**  **PD**  **local** | **Mean (SD) Controls**  **local** | **n**  **PD/ controls**  **web** | **Mean (SD)**  **PD**  **web** | **Mean (SD)**  **Controls**  **web** | **T (degrees of freedom)** | ***p*** |
| --- | --- | --- | --- | --- | --- | --- | --- | --- |
| Object invariance (skew level 2)* | 31 /50 | 0.66 (0.24) | 0.67 (0.24) | 23/248 | 0.63 (0.21) | 0.76 (0.23) | PD: 0.59 (57)  HC: -1.8 (26) | PD: 0.56  HC: 0.081 |
| Hidden Figures (number horses) | 31/23 | 14.3 (3.5) | 14.4 (4.3) | 49/231 | 14.8 (4.0) | 16.2 (3.9) | PD: -0.64 (71)  HC: -2.0 (26) | PD: 0.52  HC: 0.056 |
| Peripheral object recognition | 31/20 | 0.72 (0.19) | 0.74 (0.19) | 56/246 | 0.68 (0.20) | 0.80 (0.14) | PD: 1.1 (65)  HC: -1.2 (21) | PD: 0.29  HC: 0.24 |
| Biological motion (level 1)* | 22/19 | 0.70 (0.07) | 0.74 (0.14) | 41/170 | 0.69 (0.09) | 0.73 (0.1) | PD: 0.89 (52)  HC: 0.14 (20) | PD: 0.38  HC: 0.89 |
| Subjective size perception | 31/22 | -0.050 (0.03) | -0.070 (0.04) | 53/235 | -0.067 (0.04) | -0.069 (0.04) | PD: 2.45 (78)  HC: -0.14 (25) | PD: 0.016  HC: 0.89 |
| Mental rotation | 31/21 | 0.87 (0.08) | 0.85 (0.08) | 53/230 | 0.83 (0.1) | 0.85 (0.1) | PD: 2.0 (76)  HC: -0.27 (27) | PD: 0.046  HC: 0.79 |
| Visual acuity | 31/21 | 0.81 (0.2) | 0.80 (0.2) | 52/221 | 0.69 (0.3) | 0.82 (0.3) | PD: 2.3 (75)  HC: -0.51 (26) | PD: 0.03  HC: 0.62 |
| Tapping test  PD: n=95  HC: n=291 | 31/22 | 47 (11) | 45 (11) | 59/245 | 40 (17) | 53 (19) | PD: 2.45 (85)  HC: -3.1 34() | PD: 0.016  HC: 0.0039 ** |

HC, control; PD, Parkinson’s disease; s, seconds; SD, Standard deviation

** Significant after Bonferroni correction

* Main result used at each task for comparison between groups.

**Supplemental Table 3: Effects of age and gender on performance**

|  | **Effects of age on performance** | | | | | | | | | | | | | | | | | | | |
| --- | --- | --- | --- | --- | --- | --- | --- | --- | --- | --- | --- | --- | --- | --- | --- | --- | --- | --- | --- | --- |
|  | | | | | | Effect of PD adjusted for age | | | | Repeat analysis without oldest PD patients | | | | | | | | | | |
|  | | R^2^ | | *p* | | Estimate (SE) | | *p* | | N of new sample  (M/F) | | | Main effect PD | | Main effect difficulty | | Interaction | |  | |
| Object invariance | | 0.21 | | <0.0001** | | -0.072 (0.03) | | 0.0081** | | PD: n = 70 (37/33)  HC: n = 248 (56/192) | | | <0.0001** | | <0.0001** | | 0.12 | |  | |
| Biological motion | | 0.0085 | | 0.15 | | - | | - | | PD: n = 56 (29/27)  HC: n = 141 (48/141) | | | 0.021 | | <0.0001** | | 0.88 | |  | |
|  | | R^2^ | | *p* | | Estimate (SE) | | *p* | |  | | | PD | | Controls | | t (dof) | | *p* | |
| Hidden figures | | 0.079 | | <0.0001** | | 0.97 (0.5) | | 0.05 | | PD: n = 67 (35/32)  HC: n = 254 (59/195) | | | 15.2 (3.6) | | 16.1 (3.9) | | -1.7 (110) | | 0.085 | |
| Peripheral object recognition | | 0.15 | | <0.0001** | | 0.074 (0.02) | | 0.0001** | | PD: n = 74 (39/35)  HC: n = 246 (58/188) | | | 0.72 (0.18) | | 0.79 (0.14) | | -3.3 (102) | | 0.0012** | |
| Subjective size perception | | 0.0016 | | 0.47 | | - | | - | |  | | | - | | - | | - | | - | |
| Mental rotation | | 0.0057 | | 0.17 | | - | | - | |  | | | - | | - | | - | | - | |
|  | **Effects of gender on performance** | | | | | | | | | | | | | | | | | | | |
|  | |  |  | |  | |  |  | | | | | |  | |  | | Effect of PD Adjusted for gender | | |
|  | | Score in PD males (SD) | Score in PD females (SD) | | T (dof) | | *p* | Score in control males (SD) | Score in control females (SD) | | T (dof) | *p* | | R^2^ | | *p* | | Estimate (SE) | | *p* |
| Object invariance | | 0.60 (0.2) | 0.69 (0.2) | | -1.7 (68) | | 0.084 | 0.75 (0.2) | 0.75 (0.2) | | 0.032 (90) | 0.97 | | 0.010 | | 0.035 | | 0.11 (0.03) | | 0.00077 ** |
| Biological motion | | 0.69 (0.1) | 0.69 (0.1) | | 0.06 (61) | | 0.95 | 0.77 (0.1) | 0.72 (0.1) | | 2.6 (69) | 0.01 | | 0.011 | | 0.10 | | NA | | NA |
| Hidden figures | | 13.8 (4) | 15.8 (4) | | -2.3 (71) | | 0.02 | 15.0 (5) | 16.4 (4) | | -2.0 (81) | 0.046 | | 0.045 | | <0.0001** | | 0.91 (0.5) | | 0.083 |
| Peripheral object recognition | | 0.66 (0.2) | 0.74 (0.2) | | -2.1 (80) | | 0.037 | 0.78 (0.1) | 0.80 (0.1) | | -0.87 (92) | 0.39 | | 0.037 | | 0.00042 ** | | 0.085 (0.02) | | <0.0001 ** |
| Subjective size perception | | -0.059 (0.03) | -0.063 (0.03) | | 0.6 (74) | | 0.55 | -0.061 (0.05) | -0.071 (0.04) | | 1.67 (93) | 0.099 | | 0.015 | | 0.022 | | -0.0054 (0.005) | | 0.29 |
| Mental rotation | | 0.85 (0.09) | 0.83 (0.1) | | 0.71 (68) | | 0.48 | 0.87 (0.08) | 0.85 (0.1) | | 1.8 (131) | 0.073 | | 0.0050 | | 0.20 | | NA | | NA |

PD, Parkinson’s disease; VH, visual hallucinations; SE, Standard error; dof, degrees of freedom;

** Significant after Bonferroni correction

**Supplemental table 4. Effects of disease duration and visual acuity on performance**

|  | **Effects of disease duration** | | **Effects of visual acuity on performance** | | | |  |
| --- | --- | --- | --- | --- | --- | --- | --- |
|  |  |  |  |  | Adjusted for age | |  |
|  | R^2^ | *p* | R^2^ | *p* | Estimate (SE) | *p* | |
| Object invariance | 0.002 | 0.69 | 0.015 | 0.031 | 0.023 (0.05) | 0.63 | |
| Hidden figures | 0.0007 | 0.82 | 0.0012 | 0.54 | - | - | |
| Biological motion | 0.017 | 0.31 | 0.0048 | 0.29 |  |  | |
| Peripheral object recognition | 0.015 | 0.25 | 0.081 | <0.0001** | 0.13 (0.03) | 0.00026 ** | |
| Subjective size perception | 0.022 | 0.18 | 0.00020 | 0.80 | **-** | **-** | |
| Mental rotation | 0.014 | 0.29 | 0.0037 | 0.28 | **-** | **-** | |

PD, Parkinson’s disease; VH, visual hallucinations; SE, Standard error; dof, degrees of freedom;

**Supplemental Table 5: Comparison of performance in patients with and without hallucinations**

|  | Number with VH | Number without VH | Performance in VH mean (SD) | Performance in non-VH mean (SD) | t(dof) | *p* |
| --- | --- | --- | --- | --- | --- | --- |
| Object invariance | 14 | 67 | 0.7 (0.19) | 0.63 (0.2) | 1.3 (21) | 0.2 |
| Hidden figures | 12 | 68 | 14.8 (3.9) | 14.6 (3.9) | 0.2 (15.1 | 0.84 |
| Biological motion | 9 | 54 | 0.69 (0.09) | 0.69 (0.09) | 0.073 (10.5) | 0.94 |
| Peripheral object recognition | 15 | 72 | 0.66 (0.2) | 0.70 (0.2) | -0.66 (18) | 0.51 |
| Size perception | 14 | 70 | 0.060 (0.04) | 0.061 (0.03) | 0.07 (18.4) | 0.94 |
| Mental rotation | 15 | 69 | 0.83 (0.1) | 0.85 (0.09) | -0.64 (18.1) | 0.53 |

**Supplemental table 6: Repeat analyses with missed trials removed**

| Task | Main effect Parkinson’s | Main effect level | Interaction |  |
| --- | --- | --- | --- | --- |
| Object invariance | <0.0001** | <0.0001** | 0.047 |  |
| Biological motion | 0.013 | <0.0001** | 0.77 |  |
|  | Parkinson’s  Mean (SD) | Controls  Mean (SD) | T (dof) | *p* |
| Peripheral object recognition | 0.74 (0.17) | 0.82(0.12) | -4.0 (123) | 0.0001** |

** Significant after Bonferroni correction

**Supplemental Table 7: Number of trials with no responses in hierarchical modelling analysis**

| Task | PD  Mean number trials with no response (SD) | Controls  Mean number trials with no response (SD) | t(df) | *p* |
| --- | --- | --- | --- | --- |
| Object invariance | 0.31 (0.6) | 0.16 (0.7) | 1.8 (118) | 0.071 |
| Peripheral object recognition | 1.6 (3.4) | 08 (1.7) | 2.1 (102) | 0.038 |
| Biological motion | 0.21 (0.5) | 0.19 (0.4) | 0.23 (98) | 0.23 |

**Supplemental Table 8: Significance of the differences between people with Parkinson’s disease and Controls for Perceptual sensitivity (d’) and Criterion (c)**

| P_θ_ for each task | Perceptual sensitivity (d’) | Criterion  (c) |
| --- | --- | --- |
| Object invariance | 1** | 0.83 |
| Peripheral object | 0.998** | 0.35 |
| Biological motion | 0.96** | 0.65 |

** Significant (high levels of P_θ,_ close to 1)

**Supplemental Table 9: Mean, standard deviation and 97.5% quantiles for each group-level parameter from the signal detection theory model described in the main text.**

$\mu_{c}$ and $\mu_{d'}$ refer to the mean of the group-level posterior over criterion and *d’,* respectively. $\sigma_{c}$ and $\sigma_{d'}$ indicates the dispersion of these posteriors (the inverse of the precision parameters described in Methods, where $\sigma=1/\surd\lambda$). Parameters are separated by condition, in people with PD and in controls.

|  | PD | | | Controls | | |
| --- | --- | --- | --- | --- | --- | --- |
| Object invariance | Mean | SD | 97.5% quantile | Mean | SD | 97.5% quantile |
| $\mu_{c}$ | 0.043 | 0.060 | 0.16 | -0.042 | 0.055 | 0.066 |
| $\mu_{d'}$ | 0.93 | 0.14 | 1.21 | 1.93 | 0.14 | 2.22 |
| $\sigma_{c}$ | 0.15 | 0.10 | 0.39 | 0.53 | 0.079 | 0.69 |
| $\sigma_{d^{'}}$ | 0.47 | 0.27 | 1.01 | 1.27 | 0.16 | 1.59 |
| Peripheral Object | PD | | | Controls | | |
| $\mu_{c}$ | -0.50 | 0.070 | -0.37 | -0.47 | 0.037 | -0.40 |
| $\mu_{d'}$ | 1.96 | 0.15 | 2.28 | 2.47 | 0.80 | 2.63 |
| $\sigma_{c}$ | 0.47 | 0.062 | 0.60 | 0.39 | 0.037 | 0.46 |
| $\sigma_{d^{'}}$ | 1.13 | 0.13 | 1.42 | 0.81 | 0.075 | 0.96 |
| Biological motion | PD | | | Controls | | |
| $\mu_{c}$ | -1.21 | 0.11 | -1.03 | -1.27 | 0.075 | -1.13 |
| $\mu_{d'}$ | 1.53 | 0.18 | 1.90 | 1.92 | 0.12 | 2.18 |
| $\sigma_{c}$ | 0.34 | 0.15 | 0.61 | 0.62 | 0.067 | 0.76 |
| $\sigma_{d^{'}}$ | 0.20 | 0.15 | 0.59 | 0.33 | 0.22 | 0.77 |

**Supplemental Figure 1**

**Online assessment of visual acuity.** The letter ‘E’ was shown at fixation, surrounded by 4 outward facing arrows. Participants clicked on the arrow beside the open side of the ‘E’. The size of the ‘E’ became smaller with correct clicks, and larger with incorrect clicks (see Methods, 20 repeats). The mean size of the last 4 ‘E’s (in acuity units) was used to estimate the participant’s acuity. A calibration procedure preceded these visual tasks, whereby participants inputted measurements of distance from the screen, and size of an on-screen bar.
